# Japanese version of the Dermatology Life Quality Index: validity and reliability in patients with acne

**DOI:** 10.1186/1477-7525-4-46

**Published:** 2006-08-03

**Authors:** Natsuko Takahashi, Yoshimi Suzukamo, Motonobu Nakamura, Yoshiki Miyachi, Joseph Green, Yukihiro Ohya, Andrew Y Finlay, Shunichi Fukuhara, the Acne QOL Questionnaire Development Team

**Affiliations:** 1 Institute for Health Outcomes and Process Evaluation Research, Tokyo, Japan; 2 Department of Physical Medicine and Rehabilitation, Tohoku University Graduate School of Medicine, Sendai, Japan; 3 Department of Dermatology, Graduate School of Medicine, Kyoto University, Kyoto Japan; 4 Graduate School of Medicine, University of Tokyo, Tokyo, Japan; 5 Division of Allergy, National Center for Child Health and Development, Tokyo, Japan; 6 Department of Dermatology, Wales College of Medicine, Cardiff University, Cardiff, UK; 7 Department of Epidemiology and Healthcare Research, Graduate School of Medicine and Public Health, Kyoto University, Yoshida-Konoe-cho, Sakyo-ku, Kyoto 606-8501, Japan

## Abstract

**Background:**

Patient-reported quality of life is strongly affected by some dermatologic conditions. We developed a Japanese version of the Dermatology Life Quality Index (DLQI-J) and used psychometric methods to examine its validity and reliability.

**Methods:**

The Japanese version of the DLQI was created from the original (English) version, using a standard method. The DLQI-J was then completed by 197 people, to examine its validity and reliability. Some participants completed the DLQI-J a second time, 3 days later, to examine the reproducibility of their responses. In addition to the DLQI-J, the participants completed parts of the SF-36 and gave data on their demographic and clinical characteristics. Their physicians provided information on the location and clinical severity of the skin disease.

**Results:**

The participants reported no difficulties in answering the DLQI-J items. Their mean age was 24.8 years, 77.2% were female, and 78.7% had acne vulgaris. The mean score of DLQI was 3.99(SD: 3.99). The responses were found to be reproducible and stable. Results of principal-component and factor analysis suggested that this scale measured one construct. The correlations of DLQI-J scores with sex or age were very poor, but those with SF-36 scores and with clinical severity were high.

**Conclusion:**

The DLQI-J provides valid and reliable data despite having only a small number of items.

## Background

Of the many skin conditions that are externally visible, acne is probably the most common. A survey of Japanese students from elementary school through university revealed that 58.6% were suffering from the condition, and that 93.3% of the university students had suffered from acne at one time [1]. In a survey conducted in Great Britain, 95% of 16-year-old males and 83% of 16 year-old females had acne [2]. While almost all young people experience acne at least once, it has been reported that 47% of those in their 20s and 30% of those in their 30s had acne [3]. Numerous studies have revealed that acne is not simply a problem of the young: it also afflicts people of many ages, and it affects daily life. Mallon et al. used the Short Form 36 (SF-36), a generic quality of life (QOL) scale, to compare the QOL of acne patients to that of patients with asthma, epilepsy, diabetes, back pain, arthritis, and coronary heart disease [4]. They found that the SF-36 mental health score of patients with acne was much lower than those of all the others, even after adjusting for age and sex. Moreover, the SF-36 social functioning score for patients with acne was also lower than that of all the other patients except those with coronary heart disease. These findings show that the impact of acne on QOL can be as great as that of severe and even life-threatening diseases.

The impact of acne on QOL has been documented also in Japan. Studies using the Japanese version of the Skindex-16 [5] indicated, for example, that frequent clinic visits and decorative cosmetics can improve QOL [6,7]. Still, less attention has been given to the QOL of patients with acne in Japan than in other countries. In looking for instruments for measuring QOL among dermatology outpatients, we sought one that was short enough to impose only a minimal burden on the patients, one that gives information about areas of interest that are not covered by other measures, and one that would allow data collected in Japan to be compared with those collected in many other countries [8]. We therefore developed a Japanese version of the Dermatology Life Quality Index (DLQI) created by Finlay et al. [9,10]. The DLQI is short, and it has been used internationally for more than 10 years in the assessment and comparison of QOL of patients with acne, eczema, psoriasis, and other dermatologic conditions. One study comparing various QOL measures found only weak correlations between the DLQI score and the Skindex "symptoms" subscale, which indicates that the two scales provide information about different domains of QOL [11]. The DLQI includes questions about symptoms and feelings, daily activities, leisure, work and school, personal relationships, and treatment. DLQI scores can range from 0 to 30, and higher scores indicate poorer QOL. The DLQI is available in English, Danish [7], Norwegian [12], Spanish [13], etc. [10]. We first translated the DLQI into Japanese and adapted it for use in Japanese subjects, and then used psychometric methods to study the validity and reliability of the Japanese version in patients with acne. This research was approved by the Ethics Committee of the Public Health Research Foundation.

## Methods

### Translation and cultural adaptation

Permission to create a Japanese version was obtained from the authors of the original DLQI. Two native Japanese translators independently translated the original English version into Japanese. Then, a discussion was held on the specifics of the translation based on the two translations, and a single Japanese version was created. A translator whose native language was English then translated the Japanese version back into English. Based on the back translation, discussions were held with the original author, and the Japanese version was finalized.

Since our objective was to develop a tool for measuring the QOL of acne patients, the phrase in the original "because of your skin" was deemed to be too vague because it could include effects of skin conditions other than acne. It is further hoped that the DLQI-J would be used for other specific conditions besides acne. Thus, it was decided to change the expression "because of your skin" to "because of your (disease name)" to sharpen the focus on the burden of a specific disease. The questionnaire together with the changes described above was then submitted to the authors of the original questionnaire, and they approved the Japanese version. The expression "because of your acne" was used in our research described herein.

Pilot testing on 10 patients with acne was then conducted using the Japanese version developed as described above, and the content validity and language were assessed.

### Validation study

A total of 204 patients who were at least 16 years old and had come for treatment of acne on an outpatient basis to Departments of Dermatology at 9 hospitals were enrolled in the validation study, regardless of their treatment history or their current method of treatment.

The investigator explained the purpose of the research and how the survey was to be conducted, based on an informed consent form that was provided to the subject during the outpatient examination. After the study was explained, the consent of the subject to participate in the study was obtained. The subjects were then asked to immediately fill out the questionnaire, and it was collected as soon as they finished. Participants in 2 of the 9 hospitals were given another copy of the questionnaire for retesting to take home. These subjects filled out the second questionnaire 3 days later and mailed it to the data center.

The survey was done with a self-administered questionnaire that included the DLQI-J (10 items), five subscales from the SF-36 measuring QOL domains thought to be important to patients with dermatologic conditions (role-physical, role-emotional, social functioning, mental health, and vitality) [15-17], and questions about demographic and clinical characteristics (sex, age, and perceived severity of acne symptoms). As the index of perceived severity of acne symptoms, the patients answered 7 questions about blackhead acne, whitehead acne, acne scars, etc. on a scale from "none at all" (1 point) to "very severe" (5 points). The possible total scores on that scale thus ranged from 7 to 35. The retest contained only the DLQI-J. Information obtained from physicians included the name of the patient's condition, presence of complications, duration of disease, type of acne, and treatment history. In addition patients given only topical medications and vitamins were categorized as having clinically mild disease, those given non-topical medications (including non-topical antibiotics) were categorized as having clinically moderate disease, and those in whom permanent scarring was expected were categorized as having clinically severe disease.

Item analysis was done to determine whether the percentage of missing data for each item exceeded 10% and whether the responses were skewed. As in the scoring of the original English version, if a patient did not indicate an answer, the response was coded as 0 (the same code used to indicate "does not apply"). Any patient who did not answer at least 1 item was considered to be a nonresponder. Internal consistency reliability was assessed with Cronbach's alpha coefficient. Test-retest reliability was evaluated with Pearson's and the intra-class coefficients for correlation between the first test and the retest. Construct validity was investigated by testing for unidimensionality (principal components analysis). or higher. Finally, concurrent validity was studied with SF-36 scores, and criterion-based validity was studied with clinical severity and demographic variables.

## Results

### Translation and pilot test

A pilot test was conducted in patients with acne, and no problems were found with regard to content validity.

The terms "social activity" in item 5, "partner" in item 8 and "sexual difficulties" in item 9 were found to be difficult to translate from English into Japanese. Rather than "direct" translations, more natural and descriptive wordings were used in Japanese to ensure easy understanding and avoid needless confusion.

Items 2 through 9 ask about the effects of skin disease on daily functioning, etc., but item 1 asks directly about dermatologic symptoms themselves, and item 10 asks about therapy. Therefore, for items 1 and 10 we did not use the Japanese name of the disease, but instead the Japanese word for "skin". Two Japanese words correspond closely to the concept of "skin": *hada *mainly refers to the skin of the face, and *hifu *is a slightly more technical term that, strictly speaking, corresponds to skin in general. Since the questionnaire is expected to be used not only in patients with acne but also in those with other dermatological conditions, the more general term (*hifu*) was used to avoid confusion.

Finally, approval of the back translation and layout was obtained from the authors and the DLQI-J was completed.

### Subject characteristics

Data were analyzed from 197 subjects who responded to the DLQI-J (44 took the retest). In addition, associations between the DLQI-J and the clinical data obtained from physicians were evaluated for 196 subjects.

The mean age of subjects was 24.8 years (SD: 7.4); 77.2% (152) were females and 22.8% (45) were males. The most common type of acne was acne vulgaris (78.7%, 155), followed by acne pustulosa (11.7%, 23), and acne conglobata by (4.6%, 9). (Table 1)

**Table 1:** Characteristic of patients

	**Characteristics**	
Mean age (years)		24.8 (7.4)
Females (%)		77.2
Type of acne (%)	Acne vulgaris	78.7
	Acne pustulosa	11.7
	Acne conglobata	4.6
Duration of acne (months)		47.8
Chronic condition (%)		44.2
Severity (%)	Mild	59.3
	Moderate	37.0
	Severe	3.7
Type of treatment	Oral	
	Antimicrobial (%)	45.2
	Vitamin(except vitamin A, %)	52.8
	Other (%)	10.7
	Topical	
	Antimicrobial (%)	67.0
	Nonsteroidal anti-inflammatory (%)	9.1
	Other (%)	20.8

Data from 204 patients who were at least 16 years old and had come for treatment of acne on an outpatient basis to Departments of Dermatology at 9 hospitals.

### Item analysis

The percentage of missing values among the 10 DLQI-J items ranged from 0.5% to 4.6%. In response to item 7 ("Over the last week, has your skin prevented you from working or studying?") 95.4% of the subjects answered "No".

Scores were computed by assigning 3 points to "very much", 2 points to "a lot", 1 point to "a little", and 0 points to "not at all". The means, standard deviations, maximum values, and minimum values are shown in Table 2.

**Table 2:** Descriptive statistics of DLQI-J (N = 197)

		Mean	SD	minimum	maximum	percentile		
						25	50	75
DLQI-J^a)^	Total Score	3.99	3.99	0	20	1.00	3.00	6.00
	Symptom and feeling	2.03	1.45	0	6	1.00	2.00	3.00
	Daily activities	0.58	1.04	0	6	0.00	0.00	1.00
	Leisure	0.54	0.94	0	6	0.00	0.00	1.00
	Work and School	0.25	0.53	0	3	0.00	0.00	0.00
	Personal relationships	0.25	0.64	0	4	0.00	0.00	0.00
	Treatment	0.34	0.62	0	3	0.00	0.00	1.00

English version^b)^	Acne patient Total score	4.3	3.1	0	11	-	-	-

a) Symptom and feeling: items 1 and 2, Daily activities: items 3 and 4, Leisure: items 5 and 6, Work and School: item 7: Personal relationships, items 8 and 9, Treatment: item 10

b) From a validation study done in the UK.^9)^

### Reliability

Cronbach's alpha for the DLQI-J was α = 0.83. Exclusion of any one of the 10 items did not increase α by more than 0.01. For the test-retest data, Pearson's and intra-class correlations are shown in Table 3.

**Table 3:** Test-retest reliability correlation coefficients

	Correlation coefficient*	ICC**
Total Score	0.91	0.90
Symptom and feeling	0.65	0.62
Daily activities	0.80	0.78
Leisure	0.81	0.81
Work and School	0.56	0.57
Personal relationships	0.64	0.64
Treatment	0.49	0.50

N = 44; *Pearson's correlation coefficient; ** Intraclass Correlation Coefficient

### Validity

In the scree plot from the principal components analysis, the eigenvalue of the first component was 4.26, and the eigenvalue of the second component was 1.02. Loadings of each item on the first component are shown in Table 4.

**Table 4:** Factor loadings of DLQI-J items

**Item number**	**Item content**	**Loading on component 1**
5	Social activities	.863
3	Shopping/home	.736
2	Embarrassment	.717
7	Working or studying	.668
10	Treatment difficulties	.659
4	Clothes	.649
8	Interpersonal problems	.649
6	Sport	.583
1	Itchy, sore, painful or stinging skin	.485
9	Sexual difficulties	.397
	Contribution	42.6%

Results of principal components analysis (N = 197).

For concurrent validity, the correlations between the DLQI-J score and scores on the "social functioning", "role emotional", "mental health", and "vitality" subscales of the SF-36 were all greater than 0.40 (Table 5).

**Table 5:** Correlation between DLQI-J and SF-36 domains

**SF-36**	**DLQI-J**
Role-physical	-0.33
Vitality	-0.42
Mental health	-0.48
Social functioning	-0.49
Role-emotional	-0.49

N = 197

Men and women did not differ with regard to mean DLQI-J score (p = 0.98). Age adjustments were not done, because mean age did not differ significantly between men and women.

The correlation between age and DLQI-J scores was r = -0.14. The subjects were then divided into three age groups: teens, 20s, and all others. No differences were found among the three age groups, by one-way analysis of variance (p = 0.25).

**Figure 1:**
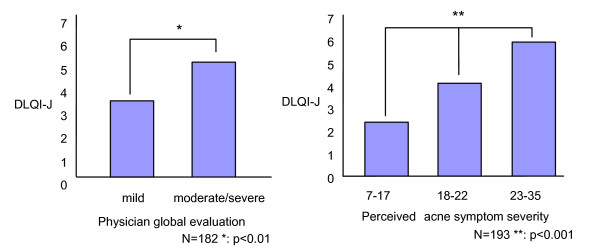
**DLQI-J mean score and acne severity**. Physicians categorized each patient's acne as mild, moderate, or severe. Data from those with acne rated as "moderate" and "severe" were combined into one group, because the number rated as "severe" was very small. The patients rated the severity of their own acne on a scale ranging from 7 to 35.

According to their physicians' evaluations of their acne, the subjects were divided into two "severity" groups: severe or moderate, and mild, and the mean DLQI-J scores for the groups were compared. The mean DLQI-J score of moderate-or-severe group was significantly higher than that of mild group (Figure 1). Acne symptoms were also converted to a global score (7 to 35 points) and the subjects were divided into three groups based on the distribution of the symptom score. Subjects with more severe acne symptoms had higher DLQI-J scores (Figure 1).

## Discussion

We developed the DLQI-J and used psychometric methods to study its validity and reliability in patients with acne. Because it comprises only 10 items, one might think that the DLQI-J is imprecise. However, the results of the present analyses show that it can provide results that are both reliable and valid. The combination of high precision and a small number of items makes this scale particularly well-suited to clinical research and epidemiological surveys.

### Total score for DLQI-J

The eigenvalue of the first component was much higher than those of the other components, and the percentage of the total variance explained by the first component was high (43%), which indicate that the DLQI-J can be treated as a unidimensional scale. The Norwegian version of the DLQI was also found to be unidimensional [13]. In addition, the total scores were generally more widely distributed than the subscale scores (Table 2), and test-retest reproducibility of the total scores was better than that of the subscale scores (Table 3). These psychometric findings lead us to recommend that only the total scores be used. We see no need to compute subscale scores. In acne patients who completed the original English version of the DLQI, the mean score was 4.3 (SD: 3.1) [9,10]. The mean score in Japan was almost the same as that in United Kingdom.

### Test-retest reliability

Test-retest reliability of the DLQI-J was slightly less than that of the original English version(Table 3) [9], but was nonetheless considered to be sufficient.

### Correlations between items

The principle component analysis revealed that the correlation between the "sexual difficulties" item and the total score was weak. Even though the question was limited to "because of acne", some of the subjects, particularly those in their teens and 20s, might have answered with reference to factors other than acne. Whatever its cause, this phenomenon was limited and we believe it does not compromise the validity of DLQI-J scores.

### SF-36

Among the SF-36 subscales measured, the weakest correlation was between DLQI-J scores and role-physical scores. As might be expected, the respondents apparently did not attribute effects of acne on role functioning to the purely physical aspects of the acne [4].

### Sex

No relationship was found between sex and the DLQI-J. This was also the case with the original English version of the questionnaire (in a study that including people being treated for skin conditions other than acne) [9]. In a survey of people with acne who were selected randomly from the general population, women's DLQI scores were higher than men's (indicating poorer QOL among the women) but the difference was not statistically significant [4]. We studied only patients who were undergoing treatment, and found that the impact on daily life did not differ between men and women. Differences between the sexes might be found if people suffering from acne but not receiving treatment are studied.

### Age

We found no correlation between age and DLQI-J scores, but Lasek reported an inverse correlation between the age of patients with acne and their QOL [18]. The difference between our results and Lasek's might be attributable to the large proportion of subjects in our study whose age was less than 30. Age-based differences should be studied in larger samples with a wider age range.

### Severity

DLQI-J scores were found to be correlated with clinical severity. This was true for both physician-reported severity and patient-reported severity. However, in many cases patients' and physicians' ratings of severity differed greatly. The patients' ratings were dichotomized between severity scores of 19 and 20, and for the physicians' ratings the "moderate" and "severe" categories were combined. Physician-patient discrepancies were found in 61 cases (34% of the total). In 16 cases (9%) the physician rated the acne as moderate or severe while the patient gave it a low rating on the severity scale, and in 45 cases (25%) the physician rated the acne as mild while the patient gave it a high rating on the severity scale. Further research is needed to determine whether such patients and physicians are aware that their judgments about acne severity are discrepant, and the causes of those discrepancies.

### Generalizability

Population-based studies are needed to find out the extent to which the results reported here can be generalized. Only 11.8% of people with acne are undergoing treatment for acne [1], and quality of life may differ between those who seek medical care and those who do not. Another topic for future study is the utility of the DLQI-J in patients with other dermatologic conditions (tinea pedis, urticaria, etc.).

## Conclusion

The DLQI-J provides valid and reliable data despite having only a small number of items. Overall, our psychometric assessment of the DLQI-J indicates that this scale is useful as a measure of disease-specific QOL in patients with acne. The user's manual for the DLQI-J [19] is available via .

## The Acne QOL Questionnaire Development Team

Norihisa Matsuyoshi (Kyoto National Hospital), Ken-ichi Toda (Kitano Hospital), Atsuko Takeda (Takeda Hospital), Miho Matsui (Takeda General Hospital), Setsuko Kondo (Otowa Hospital), Setsu Kobayashi (Kyoto-Katsura Hospital), Toshiyuki Kitajima (Uji-Tokushukai Hospital), Yukari Hattori (Shiga Medical Center for Adults).

## Competing interests

This research was supported by the public health research foundation.

## Authors' contributions

Takahashi N carried out the analysis and interpretation of data, drafted and revised this article. Suzukamo Y assumed the coordination and design of this study, training interviewers and interpretation of data. Nakamura M, Miyachi Y and the Acne QOL Questionnaire Development Team contributed in the design of this study and acquisition of data. Green J interpreted the data, edited this article for language and commented on the paper. Finlay AY and Fukuhara S contributed in the concept and design of this study, interpretation of the data, and revising the article critically for important intellectual content.
